# A dataset of a whole network to investigate healthy and unhealthy behaviors in an Italian Community

**DOI:** 10.3389/fsoc.2026.1743234

**Published:** 2026-06-11

**Authors:** Simone Sarti, Marco Terraneo, David Consolazio

**Affiliations:** 1 Università degli Studi di Milano, Milan, Italy; 2 Università degli Studi di Milano-Bicocca, Milan, Italy

**Keywords:** data protocol, health behaviors, health inequalities, network dataset, whole network analysis

## Abstract

**Introduction:**

The data protocol presented here stems from a study investigating the role of social networks in shaping health-risk behaviors in a small Lombard municipality (Italy) characterized by typical sociodemographic features.

**Methods:**

A quantitative survey was conducted on all residents aged 18–75, complemented by detailed mapping of intra- and extra-community social ties. Data collection employed structured questionnaires covering sociodemographics, health-risk behaviors, and social relationships, ensuring high data reliability as confirmed by comparison with official ISTAT statistics. Face-to-face interviews, combined with careful ethical procedures and respondent incentives, facilitated participation and minimized bias.

**Results:**

The research achieved 578 interviews, representing a 66.3% response rate among eligible residents.

**Discussion:**

The study demonstrates the feasibility of reconstructing comprehensive social networks in small communities, providing a robust empirical contribution for analyzing the clustering and transmission of health-risk behaviors. The data collected could offer valuable insights for targeted public health interventions aimed at improving health outcomes and reducing inequalities.

## Introduction

The survey described here represents the extensive fieldwork phase of a Progetto di Rilevante Interesse Nazionale (PRIN, Project of National Relevance), which was selected under the 2022 call of the Italian Ministry of University and Research and formally funded in October 2023. The project title is HEALING—HEAlthy Lifestyles in an Italian Community. The website, hosted by the University of Milan, is at: https://prinhealing.unimi.it/en.

The project was recognized as relevant within Italy's National Recovery and Resilience Plan (PNRR), addressing two key national priorities: health and research and innovation. In particular, the project aligns with one of the PNRR's “Health Missions,” which promotes the establishment of Community Health Houses (Case della Salute). The study's findings are expected to contribute to this policy goal by identifying effective ways to reduce health inequalities and enhance community wellbeing through relational and network-based interventions.

## Background and theoretical rationale

Health-related practices have long been a central topic in the social sciences, where lifestyles and risk behaviors are understood not simply as individual choices, but as socially patterned practices embedded in broader structures of inequality. Research on the social determinants of health has shown that socioeconomic resources, education, occupation, and family background shape the distribution of health risks across populations (Wilkinson and Marmot, 2003; Phelan et al., 2010). From this perspective, unhealthy behaviors such as smoking, excessive alcohol consumption, poor diet, physical inactivity, and other risk-related practices are unevenly distributed because individuals differ in their access to material, cultural, and informational resources.

At the same time, health-related behaviors are also relationally embedded. They are learned, reinforced, normalized, or discouraged through everyday interactions within families, friendship groups, workplaces, neighborhoods, and other contexts of sociability. This relational dimension suggests that lifestyles should not be treated only as attributes of isolated individuals, but also as practices that may cluster within connected groups and shared social environments.

Social network analysis provides a suitable framework for examining this dimension. By focusing on ties among actors, network approaches make it possible to study how relational proximity, network position, and community structure are associated with behavioral similarities and potential processes of influence or diffusion. Previous research has shown that several health-related behaviors, including smoking, alcohol consumption, obesity, and physical activity, may display forms of clustering within interpersonal networks (Christakis and Fowler, 2008, 2013; Rosenquist et al., 2010; Aral and Nicolaides, 2017).

The dataset presented in this article was designed to contribute to this line of research by integrating individual-level survey information with relational data on social ties within a local community. Its main value lies in enabling future analyses of how socioeconomic characteristics and network embeddedness jointly relate to the distribution of health-risk behaviors.

## The research question

The overarching objective of the project that generated the dataset presented here is to investigate the role of social networks in shaping health-related risk behaviors, such as tobacco smoking, alcohol consumption, unhealthy dietary patterns, sedentary behavior and physical inactivity, and problem gambling, and lifestyle patterns within a small municipality in Lombardy (Northern Italy) characterized by “typical” sociodemographic features. Existing literature consistently identifies both structural factors, primarily education and occupation, and relational factors, especially family and friendship networks, as key determinants of health. The project aimed to advance understanding of the interaction between these structural and relational dimensions and their combined influence on health-risk behaviors.

Whereas structural factors tend to remain stable or evolve slowly over the life course, social networks are dynamic systems: ties such as friendships and acquaintances form and dissolve more rapidly than occupational or educational statuses. The study's main research questions therefore concern:

- How do structural characteristics and relational network dynamics interact in shaping health-risk behaviors within a local community?

This broader question can be articulated into a set of more specific research questions:

- To what extent do health-risk behaviors cluster within relationally connected groups of individuals? Here, “clustering” refers both to behavioral similarities among socially connected individuals and to the emergence of cohesive network communities characterized by similar lifestyle patterns.
- What is the relative contribution of structural factors (e.g., education, occupation, age, and gender) and relational factors (e.g., peer networks, friendship ties, and family connections) in explaining the distribution of health-risk behaviors? This issue may be investigated through statistical models integrating sociodemographic and network variables.
- How are health-risk behaviors transmitted or diffused through interpersonal relations? In this context, “diffusion” refers to processes of behavioral contagion, social influence, imitation, and reinforcement occurring through repeated social interaction within networks.
- The dataset was specifically designed to allow the operationalization of these concepts through the reconstruction of complete intra-community networks, the collection of egocentric external ties, and the integration of sociodemographic and behavioral information.

A detailed understanding of how static structural features interact with dynamic relational patterns can enhance the effectiveness of social health interventions, for instance by identifying influential individuals, opinion leaders, or network communities through which unhealthy practices may spread (Bot et al., 2016; Kim et al., 2015; Valente, 2010).

The project's central goal was to reconstruct the complete social networks of individuals living in a local community, in order to explore how these networks relate to health-risk behaviors. This approach is unprecedented in Italy. Internationally, the most prominent example of this kind of socioepidemiological design is the Framingham Heart Study, a longitudinal study conducted in Massachusetts that mapped entire social networks to analyze the diffusion of health-related behaviors (Zhao and Hu, 2016; Christakis and Fowler, 2013).

The study employed a mixed-methods design, integrating a large-scale quantitative survey, collecting sociodemographic, behavioral, and relational information from all community members, with a qualitative component aimed at deepening understanding of specific social profiles emerging from the quantitative analysis.

In this paper, we limit our discussion to the strictly methodological aspects of the quantitative component of the project, while only briefly outlining the qualitative component of the research.

## The quantitative survey

Over the past decades, research in social network analysis has consistently demonstrated that health-related lifestyles and consumption practices are strongly conditioned by the networks in which individuals are embedded, by peer-group dynamics, and by local contextual factors, so-called neighborhood effects (Smith and Christakis, 2008). It is therefore plausible that an individual's behavioral traits (for instance, smoking habits) are closely related to those of their immediate social environment. Accordingly, identifying not only individual practices but also the relational context in which they occur is crucial for understanding behavioral clustering and contagion.

The core activity of the project thus consists of reconstructing the entire web of social relations within a specific community, inspired by the theoretical and methodological framework of social contagion (Bevelander et al., 2018; Mercken et al., 2010; Fowler and Christakis, 2008, 2010). To accomplish this, the research team selected a small Lombard municipality with a population of about 1,500 inhabitants, small enough to allow a near-complete mapping of interpersonal interactions. This design makes it possible to observe reciprocal influences that reinforce or weaken health-related lifestyles and consumption practices.

The HEALING project focuses on several unhealthy lifestyle behaviors extensively documented in epidemiological and sociological research (Forouzanfar et al., 2015; ISTAT, 2016):

- Tobacco smoking, which remains a leading cause of preventable mortality and is causally linked to major diseases such as cancer and cardiovascular conditions;
- Alcohol consumption, associated with increased risks of chronic liver disease, cardiovascular disorders, injuries, and social problems including alcohol-related violence;
- Unhealthy dietary patterns, contributing to hypertension, high cholesterol, overweight, obesity, and elevated blood glucose levels;
- Sedentary behavior and physical inactivity, known to raise the risk of cardiovascular disease, certain cancers, and type 2 diabetes, while reducing musculoskeletal health and psychological wellbeing;
- Problem gambling, which, though less studied in epidemiology, represents an emerging behavioral health risk.

**Figure 1:**
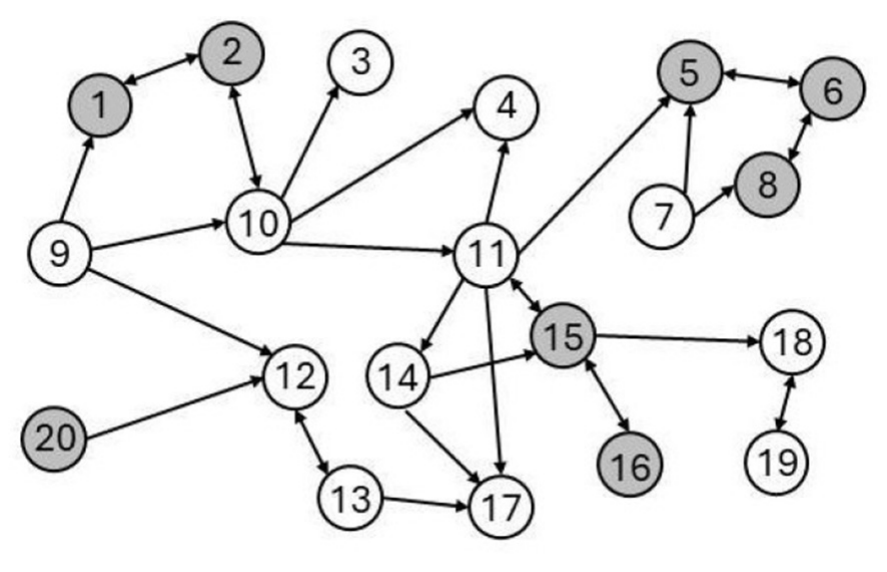
Example of a relational network.

By reconstructing both the complete local network and the external ties connecting residents to individuals outside the municipality, the study seeks to provide a comprehensive picture of how social relations mediate behavioral diffusion. An example of a network reconstruction is illustrated in Figure 1 (readapted from Valente, 2012).

In this illustrative example, gray-colored nodes represent smokers, whereas white nodes represent non-smokers. Some clusters are visible, for instance, nodes 5, 6, and 8, or the pairs 1–2 and 15–16, suggesting proximity in behavior. The goal is to observe how relational neighborhoods differ between the two groups (smokers and non-smokers) and to assess whether these configurations are associated with sociodemographic or socioeconomic attributes such as age, educational attainment, or employment status.

By accounting for both physical and virtual ties (including online contacts through platforms such as WhatsApp or Facebook), the project establishes a foundation for analyzing how behaviors known to be detrimental to health are distributed within the population and transmitted through family, friendship, and workplace networks, as well as through co-presence in shared social spaces such as cafés, schools, public areas, and churches.

## The research design

The HEALING project was organized into three main phases:

- Selection of the community in which to conduct the research;
- Implementation of a quantitative survey targeting all community members, using a structured questionnaire as the primary instrument;
- Conducting 48 in-depth interviews on a sub-sample of respondents from the previous survey.

Since administrative and social life in Italy is largely organized at the municipal level (Putnam, 1993), the study community was defined as a small municipality, allowing clear identification of all members of the community.

The selection of the municipality was based on several criteria:

- Demographic size, ensuring a manageable number of interviews compatible with the project budget while allowing identification of contacts among residents;
- A certain level of geographical isolation, to maximize the role of local ties relative to contacts outside the community;
- Administrative affiliation with the Province of Lodi, facilitating collaboration with the Health Protection Agency of Milan (ATS—Agenzia di Tutela della Salute); and
- Presence of institutions (such as primary school, parish and church youth center, pharmacy, public playground and public library) that guarantee a minimal level of social cohesion.

Specifically, the population size had to be neither too small nor too large. Municipalities with approximately 1,500 residents as of January 2024 were selected based on demographic data and preliminary cost estimates.

Geographic isolation was required to prioritize local network interactions over external contacts, so municipalities near medium/large urban centers or major transport hubs were excluded. The isolation should be considered relative, as we are not referring to small mountain villages, such as those in the Alps or the Italian Apennines, often accessible only by a single road leading to the nearest valley town, but rather to a lowland municipality with multiple connections within a well-developed road network linking neighboring towns. Nevertheless, the settlement remains cohesive and somewhat separated from those of the surrounding municipalities—a situation typical of the Po Valley.

The choice of the Province of Lodi was relevant because the project's research group had previously collaborated with ATS-Milano, which has an administrative affiliation with the province itself. The ATS-Milano (Health Protection Agency of Milan) is a regional public health authority in Italy responsible for coordinating healthcare services and implementing health policies within its jurisdiction. This cooperation enabled mutually beneficial scientific exchanges, access to aggregated ecological data, and likely increased community participation and survey response rates.

Minimal social cohesion was ensured by the presence of a parish, a primary school, a pharmacy, a central square for social events, and a library, providing basic resources to foster interpersonal relationships among residents.

**Figure 2:**
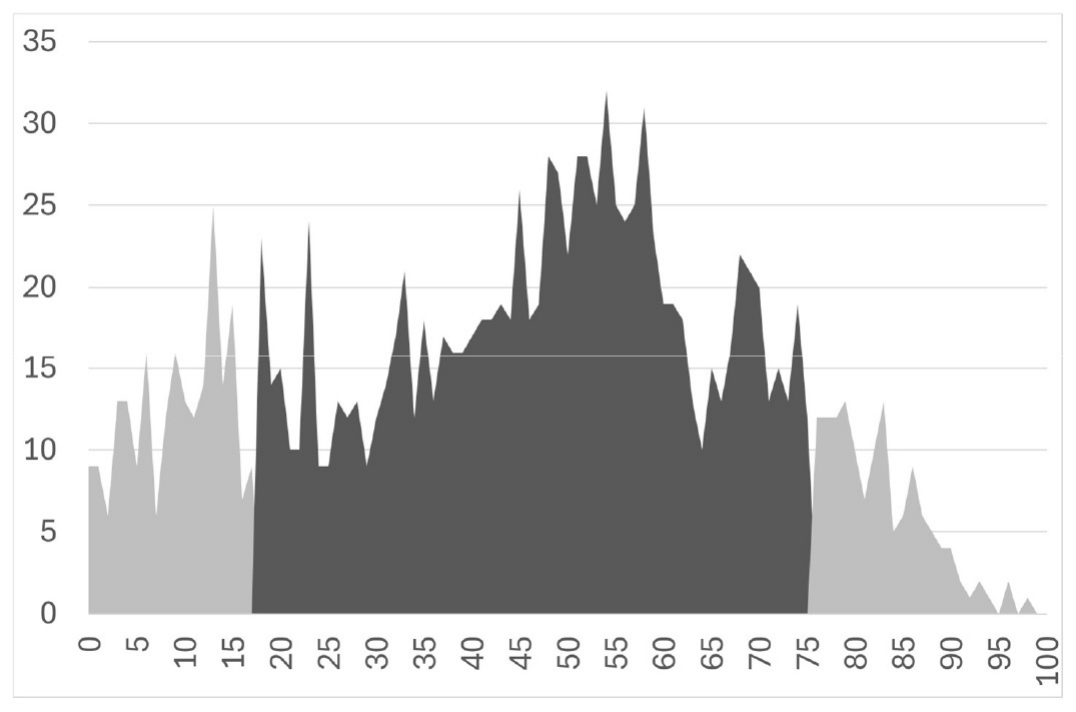
Age distribution in the municipality as of 1 January 2024.

Once the municipality was selected, a quantitative survey was conducted using a standardized questionnaire, with face-to-face interviews administered through Computer-Assisted Personal Interviewing (CAPI), collecting detailed information on socioeconomic status, health conditions, health-risk behaviors, and social relationships (see Figure 2). The target population for this extensive survey included all residents listed in the municipal electoral rolls of the selected municipality as of July 31, 2024. Data extraction from the electoral rolls was performed by municipal staff upon written request from the project's principal investigator. The electoral rolls provided each individual's name, surname, gender, date of birth, and residential address. Considering all residents aged between 18 and 75, excluding minors due to the specific nature of their social networks, typically centered on family and school, and very elderly individuals, whose networks are often markedly reduced in size and diversity, the total eligible population consisted of 923 individuals.

To ensure a satisfactory response rate and support robust statistical analysis, part of the project budget was allocated to participant incentives, consisting of 10 shopping vouchers.

After collecting sociodemographic and health-related information, the questionnaire mapped the local relational network (intra-community network) by asking respondents to freely nominate their social contacts among other residents. A “contact” was defined as any person with whom the respondent interacts or maintains a connection, including face-to-face, remote (phone), and online (e.g., Facebook, WhatsApp) contacts.

Respondents were also asked to report contacts with individuals outside the municipality (extra-community network) with whom they maintained a close relationship, such as friends, relatives, or colleagues, following a traditional egocentric network approach (name-generator method). For each extra-community contact, basic sociodemographic characteristics and selected lifestyle-related behaviors were recorded based on the respondent's (ego's) report (Chung et al., 2005).

After the quantitative survey, which administered a standardized questionnaire to all residents aged 18–75, 48 respondents who had provided their consent to be recontacted were invited to participate in in-depth interviews. The aim was to investigate, in greater detail, the health life courses (in a diachronic manner) of specific social groups identified on the basis of sociodemographic characteristics and health-risk behaviors.

The underlying rationale is to estimate average effects through statistical models applied to survey data (e.g., social gradient linking educational attainment and health behaviors), while using in-depth interviews mainly to investigate cases that diverge from these average patterns.

The profiles of the recontacted participants were constructed through different combinations of gender, age groups, socioeconomic conditions, and health-risk behaviors (for example, an older low-educated blue-collar worker reporting a healthy lifestyle, as opposed to a young highly educated professional who smokes, is sedentary, and reports unbalanced dietary habits).

Participants in the in-depth interviews received 30 shopping vouchers as an incentive.

## The questionnaire

The questionnaire is structured into four main sections, designed to collect detailed and reliable information on households, individuals, and social networks within the community. The questionnaire is provided in the Appendix.

The questionnaire has four sections, administered differently according to the type of information collected (see Table 1).

**Table 1:** Questionnaire administration.

Section	Target respondent
I—Household form	Reference person only
II—Individual socio-demographics	All adults aged 18–75 residing in the household^*^.
III—Health-related behaviors	
IV—Social network	

^*^Guests temporarily residing in the household (e.g., visiting relatives or friends) are included only in Social network mapping section if mentioned as regular contacts, but are not interviewed.

Section I—Household information (family form):

The first section collects general information on all individuals living in the same household. Here, “family” is defined in a broad sense, including all residents sharing the same dwelling. This section is administered only to a designated reference person, typically the most available, educated, or younger household member, who can manage interactions with the interviewer.

Section II—Individual sociodemographic information:

This section collects standard sociodemographic characteristics, including age, gender, educational level, occupation, and employment status, providing data necessary to determine the socioeconomic status (SES) of respondents.

Section III—Health-related behaviors:

This section focuses on lifestyle behaviors relevant to health. Questions were drawn from widely used socioepidemiological instruments, used for comparative aims at the international level, such as the PASSI survey (https://www.epicentro.iss.it/en/passi), ISTAT surveys on health conditions (https://www.istat.it/microdati/condizioni-di-salute-e-ricorso-ai-servizi-sanitari-anni-1994-2005/), and EHIS questionnaires (https://www.istat.it/en/non-categorizzato/european-health-interview-survey-ehis). Some questions also address respondents' health conditions, adopting the *Minimum European Health Module* (Cox et al., 2009), as these may influence engagement in health-risk behaviors.

More specifically, the indicators used to measure health risk behaviors are the following (details are available in the attached questionnaire):

## Tobacco smoking (from EHIS and ISTAT multipurpose survey)

- Current smoking status (Do you currently smoke?): Yes; No, but I used to smoke; No, I have never smoked.
- Age at which the respondent started smoking.
- Main type of product smoked: cigarettes; pipe and cigars; electronic cigarettes or similar.
- Number of cigarettes smoked per day (including both manufactured and hand-rolled cigarettes).

## Alcohol consumption (from ISTAT multipurpose survey)

- Frequency of consumption of wine, beer, alcoholic aperitifs, and spirits/bitters (4–6 times per week; 1–3 times per week; less than once per week; never).
- Quantity of alcoholic beverages consumed (wine, beer, alcoholic aperitifs, spirits/bitters; quantities vary by type of beverage).
- Occurrence of consuming six or more alcoholic drinks (of any kind) on a single occasion (e.g., an evening, a party, alone) in the last 12 months.

## Unhealthy dietary patterns (from ISTAT multipurpose survey)

- Frequency of consumption of: fruit (excluding industrial fruit juices, freshly squeezed juices, smoothies, and blended juices); vegetables or salad (excluding potatoes); sugary drinks (e.g., cola, orangeade, and lemonade); sweet snacks (e.g., cakes, pastries, and ice cream); savory snacks (e.g., crisps, popcorn, salted snacks, and olives).
- Types of fats used for cooking: olive oil; other vegetable oils and fats (e.g., seed oil, margarine); butter or lard.
- Attention paid to the amount of salt and/or salty foods consumed (No; Yes, reduced over time; Yes, always pay attention).

## Sedentary behavior and physical inactivity (from ISS—PASSI survey)

- Number of days in a typical week in which the respondent walks continuously for at least 10 min to move from one place to another.
- Number of days in a typical week in which the respondent rides a bicycle continuously for at least 10 min to move from one place to another.
- Number of days in a typical week in which the respondent engages in sport, fitness, or recreational physical activities (during leisure time) for at least 10 min.
- Number of hours in a typical day spent resting or sitting (excluding sleeping time).
- Physical activity at work: performing heavy work requiring considerable physical effort (e.g., construction worker, bricklayer, and farmer); walking or performing tasks requiring moderate physical effort (e.g., factory worker, waiter, and cleaner); or sitting/standing (e.g., computer work, driving, and manual work without physical effort).

## Problem gambling (from previous research on gambling: Sarti and Triventi, 2017)

- Whether the respondent spent money in the last 12 months on any type of gambling (including prize games, games with friends, games in public places, lotteries, online games, etc.). And whether the respondent spent money on online gambling.
- Frequency of spending money on gambling.
- Amount of money spent on gambling in the last month.

Section IV—Social network mapping:

The most innovative and sensitive section collects information on social relationships. Respondents are asked to list their social contacts both within the municipality (intra-community network) and outside the municipality (extra-community network), capturing interactions across family, friendship, work, and peer networks, as well as online communications (e.g., Facebook, WhatsApp). Specifically, questions posed to inter-municipal contacts included in the list concern:

- Frequent or habitual *in-person* contacts;
- Frequent or habitual *telephone/digital* contacts.
- Occasional contacts of any kind, but involving the exchange of information related to health issues.

By contrast, questions addressed to extra-municipal contacts—not included in the Resident list—concerned:

- Frequent or habitual in-person contacts (max 8).
- Frequent or habitual *telephone/digital* contacts (max 8).

The inter-municipal part of the questionnaire aims to reconstruct the respondent's network of relationships with individuals residing within the municipality, for whom personal identification is possible through the Resident List. Interviewers had access to a complete resident list, including surname, first name, date of birth, and address for each inhabitant (see Table 2). Each resident was assigned a unique anonymous code, essential for reconstructing the social network (Figure 1). For example, reasoning on fictitious data, when Mario Bianchi (b. 24/05/1973) reports a contact with Luigi Rossi (b. 18/12/1977), the interviewer matches Rossi in the Resident list and assigns the contact the unique code X008. No other personal information is recorded. Accuracy is critical to correctly reconstruct the relational network.

**Table 2:** Example of the resident list (fictitious data).

Unique code	Surname	Name	Date of birth	Address
X008	Rossi	Luigi	18/12/1973	Via Voltaire, 10
X971	Rossi	Eleonora	29/08/1989	Via Vai, 1
…	…	…	…	…

The extra-municipal part of the questionnaire aims to reconstruct the respondent's network of relationships with individuals who are not residents of the municipality, or who are not included in the Resident List. In this case, up to eight contacts are requested, following the so-called *name generator* technique. The respondent is simply asked to indicate names or nicknames for these persons, and then to provide, indirectly, information about some of their characteristics.

The wording of the questions addressed to both intra-municipal and extra-municipal contacts is reported in the Social network mapping section of the questionnaire in Appendix.

## Ethical issues

Given the nature of the information collected, particular attention was paid to ethical considerations and to ensuring transparent data collection procedures, with respondents being fully informed and confident about how their data would be used. Protecting personal data, maintaining anonymity, and ensuring participants fully understood the purpose of the study through informed consent were essential elements of the fieldwork protocol.

Informed consent was obtained in compliance with the GDPR (EU Regulation 2016/679). Interviewers provided participants with a detailed document outlining the study objectives and data handling procedures. Participants signed the consent form before the interview could proceed and were informed that they could withdraw at any time without consequence. The study received ethical approval from the University of Milan Ethics Committee on February 20, 2024, which acknowledged the balance between collecting potentially sensitive data and the social value of investigating the diffusion of health-risk behaviors within the population.

## The fieldwork

The data gathering was entrusted to DOXA in July 2024, a social and market research institute with extensive experience in survey administration. Following an extended preparation phase, the questionnaire was implemented digitally on tablets to ensure accuracy and efficiency in data collection (https://www.bva-doxa.com).

On September 22, 2024, the research team collaborated with the municipality hosting the study to participate in a local village festival as part of a community engagement strategy. During the event, informational materials about the research project and the upcoming survey were distributed to residents. In addition, posters were placed in local businesses and on municipal noticeboards to further publicize the study, as well as on the municipality's website.

Local law enforcement, including the Carabinieri and municipal police, were notified in advance to reassure residents in case of unfamiliar individuals in the area. A complete list of interviewers was shared with authorities to allow for verification if needed.

In October 2024, DOXA sent an initial postal contact letter to all households in the municipality. The envelope prominently displayed the logos of the Municipality, the University of Milan, the University of Milano-Bicocca, ATS-Milano, and DOXA, providing official recognition and enhancing trust in the project.

Following the mailing of the letters, interviewers appointed by DOXA, who had been appropriately trained through briefing sessions also attended by members of the university research team, began door-to-door visits to assess the willingness of eligible individuals to participate in the interviews. Where possible, telephone numbers and email contacts were collected in order to facilitate the scheduling of interview appointments.

Face-to-face interviews began on November 8, 2024, and continued until March 15, 2025, the date of the final interview. Interviews were conducted either at respondents' homes or, by agreement, in the AUSER (*Autogestione dei Servizi e Unione e Relazioni*) room within the Municipal Building of the Municipality, located in the center of the town. AUSER is an Italian non-profit association for volunteering and social promotion (it is legally recognized for its welfare/assistential purposes and is registered as an association of promotion of social benefit). Offering this alternative location aimed to facilitate participation, without asking respondents to admit strangers into their homes.

The data collection process presented several methodological, ethical, and logistical challenges. From an organizational standpoint, updating the list of residents to be interviewed, managing a complex questionnaire, coordinating a team of interviewers, and coping with the extended time required for face-to-face surveys demanded careful planning and resources. Ensuring the standardization of interviews was crucial to guarantee that all interviewers posed questions consistently, while the physical presence of interviewers also introduced the potential for interviewer effects, such as social desirability bias. Some questions were inherently sensitive or potentially embarrassing, requiring tactful administration.

Participant-related challenges also emerged. Some residents refused or were unavailable to participate, while others could not respond due to health issues. Additionally, older adults or individuals with lower educational levels occasionally encountered difficulties understanding the questionnaire. Finally, building trust and ensuring the safety of both respondents and interviewers required careful attention, as some participants expressed skepticism or wariness toward the research staff.

After the quantitative survey, as planned, interviewed individuals were profiled according to a range of characteristics, including gender, age, socioeconomic status, and health-risk behaviors. As discussed above, the aim was to collect detailed retrospective information on health-related life courses in a diachronic perspective (for example, identifying when respondents started or stopped smoking, the motivations underlying these behaviors, and the role played by peers or parents in shaping such choices). Participants selected for the in-depth interviews had previously provided their consent, during the initial standardized interview, to be recontacted for a more extensive qualitative interview.

Overall, 48 individuals were interviewed, and the interview transcripts were archived for further qualitative analysis.

## Data collection

By the end of fieldwork in March 2025, a total of 578 complete interviews were successfully conducted (see Table 3). Some non-responses were recorded due to various reasons. During the survey, it emerged that 51 individuals of the 923 eligible residents were no longer living in the municipality, having relocated elsewhere. These individuals were therefore excluded from the eligible population. Additionally, 69 residents could not be contacted, 221 refused to participate, and 4 individuals completed the interview but did not sign the informed consent form, leading to the destruction of their responses.

**Table 3:** Survey outcome.

Outcome	Frequency	%
Unable to interview	69	7.5
Completed interview	578	62.6
Refusal	221	23.9
Privacy screen out	4	0.4
Relocated from the municipality (*post hoc* not eligible)	51	5.5
Total	923	100

Overall, the response rate was calculated as 578 divided by 923 minus 51, resulting in 66.3%. However, it should be noted that in 50 cases, respondents did not provide information for the relational network section of the questionnaire. Excluding these individuals reduces the effective response rate to 60.6%, however, this percentage is considered adequate for protecting the estimates of many network metrics from major biases (Smith et al., 2017; Smith and Moody, 2013).

**Figure 3:**
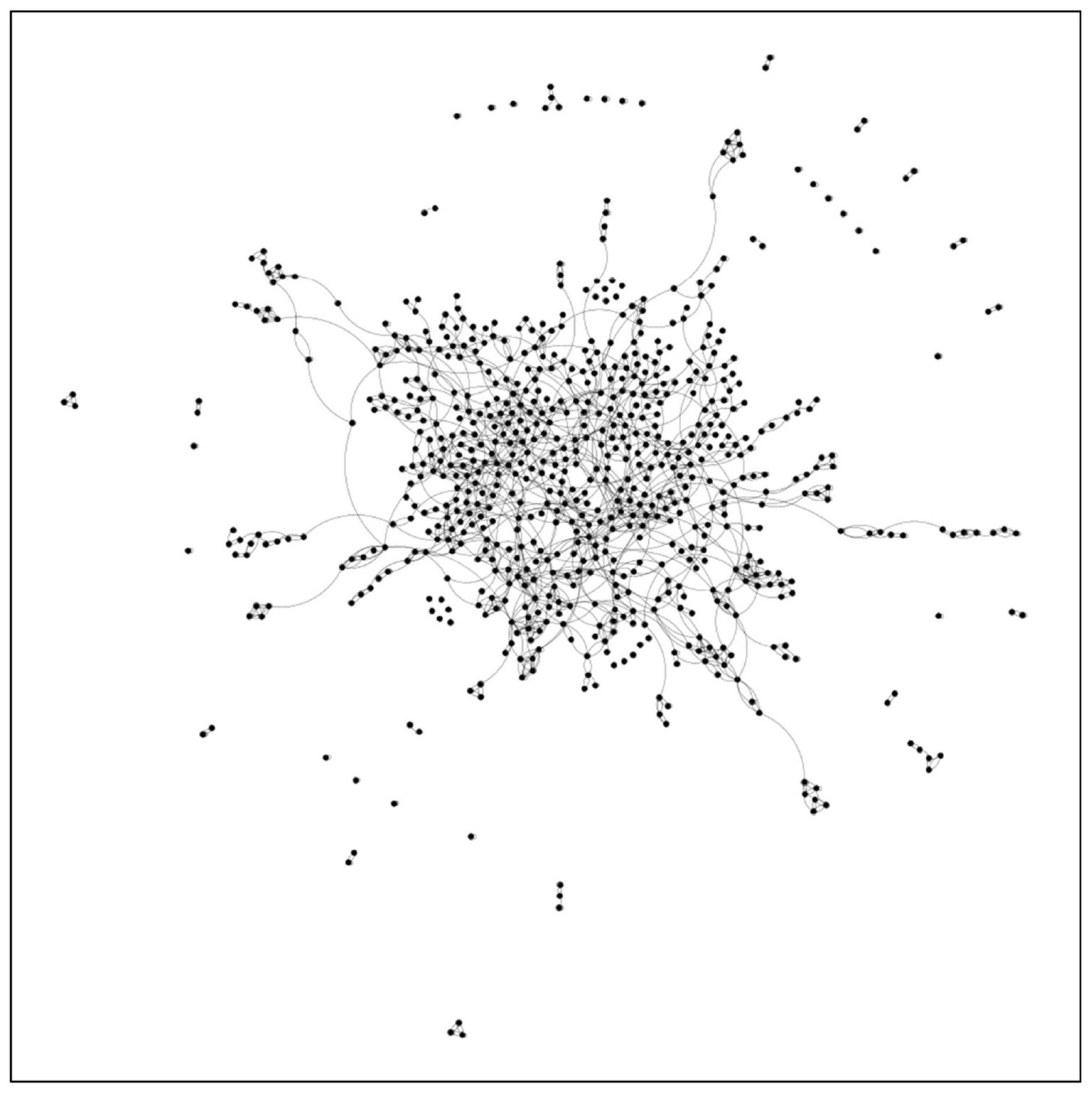
Intra-community ties (extra-community connections are not depicted).

In the final reconstruction of the social network, 709 nodes are represented, reflecting both interviewed participants and additional individuals mentioned by respondents, but not interviewed. These non-interviewed individuals appear in the dataset in a fully anonymized form, consistent with the study's privacy protocols. Consequently, the network includes approximately 80.6% of the population listed in the electoral rolls. Figure 3 illustrates the complete intra-community network, reconstructed using the Gephi software.

To assess the reliability of the collected data, descriptive analyses were performed comparing the sociodemographic characteristics of survey respondents with official statistics from ISTAT (see Table 4). Where municipal-level data were unavailable, regional data for Lombardy were used as reference. For the considered sociodemographic variables, gender, age, educational attainment, employment status, and social class (derived by a categorization of occupational positions within the labor market), as well as for key health-risk factors, the survey data showed very good correspondence with the reference population. These alignments indicate a high level of representativeness and support the validity of the collected information for further analyses of health-risk behaviors and social network dynamics.

**Table 4:** Comparison between collected data and official statistics on sociodemographic characteristics and main health-risk factors.

Variables	Data collected		Data according to official statistics by ISTAT	
	Frequency	Percentage	Municipality level	Lombardy level
Gender				
Females	300	51.9	52.3	
Males	278	48.1	47.7	
Age				
18–34	114	19.7	20.8	
35–54	202	34.9	39.5	
55–64	155	26.8	23.3	
65–75	107	18.5	16.4	
Educational attainment				
Primary or less	23	4.0		11.4
Secondary (I)	148	25.6		32.2
Secondary (II)	319	55.2		38.3
Tertiary	87	15.1		18.1
Missing	1	0.2		–
Occupation				
Not employed	211	36.5		48.3
Employed	367	63.5		51.7
Social class				
Bourgeoisie (entrepreneurs, and self-employed professionals)	35	6.1		12.0
Professionals and managers (employed)	23	4.0		
White collars (employees)	191	33.0		42.0
Manual workers	183	31.7		33.9
Autonomous	53	9.2		11.6
Missing or not assigned	93	16.1		0.5
Smoker				
No	466	80.6		79.6
Yes	112	19.4		20.4
Alcohol consumer				
No	255	44.1		50.3
Moderate	133	23.0		49.7
Strong	190	32.9		
Obese (BMI > 30)				
Normal weight	504	87.2		89.3
Obese	37	6.4		10.7
Missing	37	6.4		–

## Conclusions

The extensive survey conducted in the selected Lombard municipality has successfully reconstructed both individual-level sociodemographic profiles, health behaviors and the full relational network of the community. The study achieved a high response rate (66.3% or 60.6% if only complete interviews are considered), with the final network including approximately 80.6% of the population listed in the electoral rolls, reflecting both interviewed participants and individuals mentioned as contacts.

The survey's methodology, combining face-to-face interviews, carefully structured questionnaires, and unique anonymous identifiers for network reconstruction, ensured high data quality and reliability, with collected sociodemographic and lifestyle variables closely matching official ISTAT statistics. The project also overcame notable challenges, including logistical and ethical considerations, complex questionnaire design, and participant engagement, demonstrating the feasibility of mapping relational networks in small communities.

This contribution is consistent with a growing body of whole-network research showing that health-related behaviors are embedded in relational structures and may cluster, spread, or be reinforced through interpersonal ties. Previous studies have documented the diffusion of smoking and alcohol-related behaviors in large social networks (Christakis and Fowler, 2008; Rosenquist et al., 2010), the co-evolution of friendship networks and smoking behavior among adolescents (Mercken et al., 2010), and the usefulness of whole-population network designs for studying alcohol-related norms in local communities (Perkins et al., 2022). Moreover, network-informed interventions have shown the potential value of identifying influential actors and peer leaders for health promotion strategies (Valente et al., 2003). In this perspective, the dataset presented here may contribute to this literature by offering a rare whole-network reconstruction of an adult local community, combined with detailed information on multiple health-risk behaviors and sociodemographic characteristics.

Overall, this study provides a solid foundation for subsequent analyses of health-risk behaviors and their diffusion within social networks, offering insights that can inform targeted public health interventions and strategies to reduce health inequalities in local contexts.

## Data Availability

The datasets presented in this study can be found in online repositories. The names of the repository/repositories and accession numbers can be found below: https://doi.org/10.13130/RD_UNIMI/6BPYIJ.
