# Supplementary material for: A dataset of a whole network to investigate healthy and unhealthy behaviors in an Italian Community
Source: Front Sociol. 2026 Jun 11;11:1743234. doi: 10.3389/fsoc.2026.1743234 (PMC13293905; doi:10.3389/fsoc.2026.1743234)
Supplement: Supplementary file 1 [file Data_Sheet_1.pdf]

Finanziato dall'Unione europea
NextGenerationEU

QUESTIONNAIRE PRIN "HEALING"

VERSION - 4 November 2024

Simone Sarti, Marco Terraneo, David Consolazio, Roberta Gallina
University of Milan and University of Milano-Bicocca

Contacts:

simone.sarti@unimi.it marco.terraneo@unimib.it

Target Population

All individuals listed in the electoral registers (aged 18 to 75 years).

Available Data in the electoral registers:

- Surname
- First name
- Date of birth
- Residential address

Coding System for Electoral Register of Municipality of XXX

Unique Code	Surname	First Name	Date of Birth	Address
X0033	Mario	Rossi	18/12/1973	Via Torino, 10 – COMUNE
…	…	…	…	…

Note: The unique code is randomised.

Questionnaire Sections

I. Household Roster
II. Individual Socio-demographic Information
III. Individual Health-related Behaviours
IV. Individual Social Network

SECTION I – HOUSEHOLD ROSTER

To be completed by the Reference Person (RP).

All individuals living in the household unit must be included.
In the case of guests who are not officially resident in XXX but are temporarily staying in the household (e.g. visiting relatives, friends), these individuals, if mentioned as non-occasional contacts, will be recorded in **Section IV** as *extra-municipal contacts*. They will not be assigned a unique code and will not be interviewed.

1. Household Members

	**Member 1 (RP)**	**Member 2**	**Member 3 (not in residents' list)**	**…**
**IDF** – Household code (a)	IA0001	IA0001	IA0001	…
**IDIF** – Intra-household code	0001	0002	0003	…
**IDA** – Unique code	X0033	X????	Serial no. 0001	…
**PRE*** – Presence/Absence				
**SEX** – Sex (M / F / Other)				
**BIR** – Year of birth				
**NAT** – Citizenship (Italian / non-Italian)				
**REG** – Region of birth (Italy) or Country of birth				
**POS** – Relationship to RP (**)	1			

(a) The household code is composed of an interviewer code (IA, IB, IC, ID, IE, IF, IG, IH, IL, IM, IN…) and a serial number assigned by the interviewer to each household interviewed.

Coding Instructions

( ) Presence/Absence*

1 – Present
2 – Present but refuses
3 – Temporarily absent (further contact attempts will be made)
4 – Absent for the entire survey period due to work
5 – Absent for the entire survey period due to study
6 – Absent for the entire survey period due to health reasons
7 – Absent for the entire survey period due to holiday
8 – Absent for the entire survey period due to other reason (specify: _ _ _ _ _ _)

( ) Relationship to RP**

1 – Reference Person (RP)
2 – Spouse or cohabiting partner of RP
3 – Parent (of RP or of spouse/partner)
4 – Son/daughter of RP
5 – Brother/sister of RP
6 – Spouse or cohabiting partner of son/daughter
7 – Grandparent of RP
8 – Grandchild of RP
9 – Other relative
0 – Other non-relative

SECTION II – INDIVIDUAL SOCIO-DEMOGRAPHIC INFORMATION

2.1 Socio-demographic Characteristics

D00 – For approximately how many years have you been residing or living in the municipality of XXX ?

1. For less than 1 year

_ _ [indicate number of years]

888. Since birth

999. [Prefer not to answer / Don't know]

D01 – Current marital status

1. Single (never married)
2. Married, or in a civil union
3. Cohabiting (more uxorio)
4. Separated (de facto or legally) or divorced
5. Widowed

999. [No answer]

D02 – Highest level of education completed

1. Doctoral degree (PhD or equivalent)
2. Integrated Master's degree / Specialist or long-cycle degree / Pre-reform Laurea
3. Bachelor's degree (3 years, first cycle)
4. University diploma (2–3 years)
5. Upper secondary school-leaving certificate (4–5 years, granting access to university)
6. Vocational qualification diploma (upper secondary, 2–3 years)
7. Lower secondary school certificate (Licenza media / Avviamento professionale)
8. Primary school certificate / Final assessment certificate
9. No formal qualification

999. [No answer]

D03 – Which of the following best describes your current employment status?

1. Employed
2. Unemployed, seeking first or new employment
3. Student, in training, intern or unpaid trainee
4. Retired (old-age or early retirement) or ceased business activity
5. Unpaid domestic work (housewife/househusband)
6. Unable to work (permanently incapacitated)
7. Other inactive person

999. [No answer]

[ASK IF EMPLOYED OR RETIRED – D03=1 OR D03=4]
D04 – Referring to your main or last occupation, was this:

1. Paid employment (employee)

As a form of dependent work:

2. Coordinated and continuous collaboration (with or without project)
3. Occasional work contract

As a form of self-employment:

4. Employer / entrepreneur
5. Self-employed professional
6. Independent worker (autonomous)
7. Assisting in a family business

999. [No answer]

[ASK IF EMPLOYEE – D04=1]
D05 – Was (or is) this a fixed-term contract?

1. No, permanent contract
2. Yes, fixed-term contract

999. [Don’t know / No answer]

[ASK IF EMPLOYEE, CO.CO.CO. OR OCCASIONAL WORK – D04 ≤ 3]
D06 – Your position was (or is):

1. Executive or senior manager
2. White-collar employee / non-manual worker
3. Blue-collar worker / craftsman / manual worker

999. [Don’t know / No answer]

[ASK IF EMPLOYEE, CO.CO.CO. OR OCCASIONAL WORK – D04 ≤ 3]
D07.1 – Was (or is) your job in the public or private sector?

1. Public sector
2. Private sector
3. Mixed public-private company

999. [Don’t know / No answer]

[ASK IF SELF-EMPLOYED – D04=4, 5, OR 6]
D07.2 – Approximately how many employees did (or do) you have?

0. Self-employed, no employees
1. 1–3 employees
2. 4–14 employees
3. 15–49 employees
4. 50 or more employees

999. [No answer]

[ASK IF EMPLOYED OR RETIRED – D03=1 OR D03=4]
D08.1 – Could you please state the name of your (current or last) occupation?
ISCO Classification (2-digit level, funnel structure: first ISCO1, then ISCO2):

1. Senior officials and managers (public and private)
2. Professionals (intellectual and scientific, university graduates)
3. Technicians and associate professionals (generally with secondary school diploma, not university degree)
4. Clerical support workers (office jobs)
5. Service and sales workers
6. Skilled agricultural, forestry and fishery workers
7. Craft and related trades workers (non-mechanised)
8. Plant and machine operators, assemblers
9. Elementary occupations (unskilled)
10. Armed forces occupations
11. Other (specify)

999.[Not answer]

D08.2 – ISCO 2-digit (ISCO-08 Structure | International Labour Organization)
For each subgroup, include the option "Other (please specify)".

1. Senior Officials and Managers (Public and Private)

- **11** Managers: Senior officials of public administration, members of the executive and legislative bodies (e.g. judges, CEOs, politicians, university rectors, etc.)
- **12** Managers: Administrative and commercial managers (public administration and commerce)
- **13** Managers: Production and specialised services managers (industry, transport, construction)
- **14** Managers: Hospitality, retail and other services managers (business and personal services, sports centres, etc.)

2. Professionals (Intellectual and Scientific – University Graduates)

- **21** Professionals: Science and engineering professionals (engineers, architects, etc.)
- **22** Professionals: Health professionals (hospital doctors, graduate nurses, etc.)
- **23** Professionals: Teaching professionals (university professors, graduate teachers, etc.)
- **24** Professionals: Business and administration professionals (financial analysts, chartered accountants, PR specialists, sales managers, etc.)
- **25** Professionals: ICT professionals (IT analysts, software developers, etc.)
- **26** Professionals: Legal, social and cultural professionals (lawyers, graduate psychologists, etc.)

3. Technicians and Associate Professionals (generally with secondary diploma, not university degree)

- **31** Technicians: Science and engineering associate professionals (IT technicians, electrotechnicians, etc.)
- **32** Technicians: Health associate professionals (dental technicians, nurses without degree, etc.)
- **33** Technicians: Business and administration associate professionals (sales agents, brokers, financial agents, office supervisors, etc.)
- **34** Technicians: Legal, social and cultural associate professionals (teachers without degree, etc.)
- **35** Technicians: ICT associate professionals (web technicians, etc.)

4. Clerical Support Workers (Office Jobs)

- **41** Clerical support workers: General clerks and office machine operators (secretaries, etc.)
- **42** Clerical support workers: Customer services clerks (counter clerks, receptionists, call centre operators, etc.)
- **43** Clerical support workers: Numerical and material recording clerks (payroll clerks, shipping clerks, etc.)
- **44** Clerical support workers: Other clerical support workers (not elsewhere classified)

5. Service and Sales Workers

- **51** Personal services workers (tourist guides, hairdressers, beauticians, cooks, etc.)
- **52** Sales workers (cashiers, shop assistants, etc.)
- **53** Personal care workers (caregivers, nursing assistants, etc.)
- **54** Protective services workers (security guards, etc.)

6. Skilled Agricultural, Forestry and Fishery Workers

- **61** Skilled agricultural workers (farmers, livestock breeders for sale)
- **62** Forestry, fishery, hunting and subsistence workers (forestry workers, subsistence farmers, fishers, hunters, gatherers)

7. Craft and Related Trades Workers (Non-mechanised)

- **71** Building and related trades workers, excluding electricians (construction workers, plumbers, etc.)
- **72** Metal, machinery and related trades workers (blacksmiths, carpenters, metalworkers, etc.)
- **73** Handicraft and printing trades workers (goldsmiths, glassmakers, luthiers, etc.)
- **74** Electrical and electronic trades workers (electricians, etc.)
- **75** Food processing, woodworking, textile and related trades workers (butchers, carpenters, tailors, shoemakers, etc.)

8. Plant and Machine Operators, and Assemblers

- **81** Stationary plant and machine operators (plastics, ceramics, glass, metal profiles, foundry, wood cutting, mechanised food production, etc.)
- **82** Assemblers (assembly line workers in machinery, vehicles, appliances, engines, etc.)
- **83** Drivers and mobile plant operators (lorry drivers, couriers, riders, crane operators, etc.)

9. Elementary Occupations (Unskilled)

- **91** Cleaners and helpers (domestic helpers, janitors, etc.)
- **92** Agricultural, forestry and fishery labourers (farmhands, etc.)
- **93** Labourers in mining, construction and transport (manual workers, etc.)
- **94** Food preparation assistants (canteen assistants, pre-packaged meal preparation, etc.)
- **95** Street and related sales and service workers (street vendors, etc.)
- **96** Refuse workers and other elementary occupations (waste collectors, porters, etc.)

0. Armed Forces Occupations

- Armed forces personnel

999. [No answer]

D09 – In which economic sector are you (or were you) employed? If you have (or had) more than one job, please always refer to your **main occupation**.

[ATECO Classification – 21 categories]

Code	Section	Economic Sector
01	A	Agriculture, forestry and fishing
02	B	Mining and quarrying
03	C+D+E	Manufacturing + Electricity, gas, steam and air conditioning supply + Water supply; sewerage, waste management and remediation activities
04	F	Construction
05	G	Wholesale and retail trade; repair of motor vehicles and motorcycles
06–11, 15	H+I+J+K+L+M+N+R+S	Services
12	O	Public administration and defence; compulsory social security
13	P	Education
14	Q	Human health and social work activities
16–17	T+U	Other (please specify)
999	–	[No answer]

Household Income [ASK REFERENCE PERSON ONLY]
D10 – Does your household's available income allow you to make ends meet…

- 1 – …With difficulty
- 2 – …With some difficulty
- 3 – …Fairly easily
- 4 – …Easily
- 999 – [Prefer not to answer / Don't know]

Individual Income [ASK ALL RESPONDENTS]
D11 – Could you please indicate which of the following categories comes closest to your own net monthly income?

- 0 – I do not receive any income
- 1 – Up to €500
- 2 – €501 to €1,000
- 3 – €1,001 to €1,500
- 4 – €1,501 to €2,000
- 5 – €2,001 to €2,500
- 6 – €2,501 to €3,000
- 7 – €3,001 or more
- 999 – [Prefer not to answer / Don't know]

2.2 Health Conditions

[Note: Minimum European Health Module]

Now we will talk about health and dietary habits.

S01 – How is your health in general?

1. Very good
2. Good
3. Fair (neither good nor bad)
4. Bad
5. Very bad

999. [No answer]

S02 – Do you have any chronic (long-standing) illness or health problem (physical or mental)? The term *long-standing* refers to illnesses or health problems which have lasted, or are expected to last, for at least 6 months.

1. Yes
2. No

999. [No answer]

S03 – For at least the past 6 months, to what extent have you been limited because of a health problem in activities people usually do? Would you say you have…

1. Severe limitations
2. Some limitations (but not severe)
3. No limitations

999. [No answer]

S04 – When was the last time you consulted your general practitioner (family doctor) about a health problem?

1. Less than 6 months ago
2. Between 6 months and less than 12 months ago
3. 12 months ago or more
4. Never

999.

SECTION III – INDIVIDUAL HEALTH-RELATED BEHAVIOURS

3.1 Weight, Height, Diet

C01.1 – Could you please indicate your current weight? *(Women who are pregnant should indicate their weight before pregnancy)*

_ _ _ kg
999. [No answer]

C01.2 – Could you please indicate your current height?

_ _ _ cm
999. [No answer]

C02 – How often do you eat fruit? *(Exclude industrial fruit juices, freshly squeezed juices, smoothies, and centrifuged juices)*

1. Once or more per day
2. 4–6 times per week
3. 1–3 times per week
4. Less than once per week
5. Never

999. [Don't know / No answer]

C03 – How often do you eat vegetables or salad? *(Exclude potatoes)*

1. Once or more per day
2. 4–6 times per week
3. 1–3 times per week
4. Less than once per week
5. Never

999. [Don't know / No answer]

C04 – How often do you consume sugary drinks (e.g. cola, orangeade, lemonade)?

1. Once or more per day
2. 4–6 times per week
3. 1–3 times per week
4. Less than once per week
5. Never

999. [Don't know / No answer]

C05 – How often do you consume sweet snacks (e.g. cakes, pastries, ice cream)?

1. Once or more per day
2. 4–6 times per week
3. 1–3 times per week
4. Less than once per week
5. Never

999. [Don't know / No answer]

C06 – How often do you consume savoury snacks (e.g. crisps, popcorn, salted snacks, olives)?

1. Once or more per day
2. 4–6 times per week
3. 1–3 times per week
4. Less than once per week
5. Never

999. [Don't know / No answer]

C07 – Which of the following fats do you most frequently use for cooking?

1. Olive oil
2. Other vegetable oils and fats (e.g. seed oil, margarine)
3. Butter or lard

999. [Don't know / No answer]

C08 – Do you pay attention to the amount of salt and/or salty foods you consume?

1. No, I do not pay attention
2. Yes, I have reduced my consumption over time
3. Yes, I have always paid attention

999. [Don't know / No answer]

3.2 Alcoholic Beverages

C09 – How often do you usually consume the following alcoholic beverages?

	**Every day (5)**	**4–6 times per week (4)**	**1–3 times per week (3)**	**Less than once per week (2)**	**Never (1)**	**999 [Don't know / Prefer not to answer]**
C09.1 Wine	5	4	3	2	1	999
C09.2 Beer	5	4	3	2	1	999
C09.3 Alcoholic aperitifs	5	4	3	2	1	999
C09.4 Spirits and bitters	5	4	3	2	1	999

[C09.5–C09.8 ASK ONLY IF CONSUMPTION ≠ 1 (Never) AND ≠ 999]
In what quantity do you usually consume the following?

- **C09.5 Wine**
  4. More than 2 glasses
  3. 2 glasses
  2. 1 glass
  1. Less than 1 glass
  999. [Don't know / Prefer not to answer]

- **C09.6 Beer (40cl glass)**
  4. More than 2 glasses
  3. 2 glasses
  2. 1 glass
  1. Less than 1 glass
  999. [Don't know / Prefer not to answer]

- **C09.7 Alcoholic aperitifs**
  4. More than 2 glasses
  3. 2 glasses
  2. 1 glass
  1. Less than 1 glass
  999. [Don't know / Prefer not to answer]

- **C09.8 Spirits and bitters**
  4. More than 2 shots
  3. 2 shots
  2. 1 shot
  1. Less than 1 shot
  999. [Don't know / Prefer not to answer]

C10 – Thinking about the last 12 months, have you ever consumed 6 or more alcoholic drinks (of any kind) on a single occasion (e.g. an evening, a party, alone, etc.)?

1. Yes
2. No

999. [Don't know / No answer]

3.3 Tobacco Consumption

C11 – Do you currently smoke?

1. Yes
2. No, but I used to smoke in the past
3. No, I have never smoked

999. [Don't know / No answer]

[ASK IF CURRENT OR FORMER SMOKER – C11=1 OR C11=2] C12 – Do you remember approximately at what age you started smoking?

- Age: ___

999. [Don't remember / No answer]

[ASK IF CURRENT OR FORMER SMOKER – C11=1 OR C11=2] C13 – What do you mainly smoke now, or what did you mainly smoke in the past? (One answer only)

1. Cigarettes
2. Pipe and cigars
3. Electronic cigarettes or similar

999. [Don't know / No answer]

[ASK IF CURRENT SMOKER OF CIGARETTES – C11=1 AND C13=1] C14 – On average, how many cigarettes do you smoke per day (including manufactured and hand-rolled cigarettes)?

- Number: _ _ _

999.

3.4 Physical and Sporting Activity, Sedentary Behaviour

C15 – On how many days in a typical week do you usually WALK continuously for at least 10 minutes to get from one place to another?

- Number of days: |__|
  1. I never do this activity
  999. [Don't know / No answer]

C16 – On how many days in a typical week do you usually RIDE A BICYCLE continuously for at least 10 minutes to get from one place to another?

- Number of days: |__|
  1. I never do this activity
  999. [Don't know / No answer]

C17 – On how many days in a typical week do you usually engage in SPORT, FITNESS OR RECREATIONAL PHYSICAL ACTIVITIES (in your leisure time) continuously for at least 10 minutes?

- Number of days: |__|
  1. I never do this activity
  999. [Don't know / No answer]

C18 – Excluding sleeping time, how many hours in a typical day do you usually spend resting or sitting? Please give an overall estimate.

- ||| hours per day
  999. [Don't know / No answer]

[ASK IF EMPLOYED – D03=1] C19 – During your work, would you say you mainly:

1. Perform heavy work requiring considerable physical effort (e.g. construction worker, bricklayer, farmer)
2. Walk or perform tasks requiring moderate physical effort (e.g. factory worker, waiter, cleaner)
3. Sit or stand (e.g. computer work, driving, manual work without physical effort)

999. [Don't know / No answer]

3.5 Gambling

C20 – In the last 12 months, have you spent money on any type of gambling? *(By "gambling" we mean prize games, games with friends, games in public places, lotteries, online games, etc.)*

1. Yes
2. No

999. [Don't know / No answer]

[ASK IF GAMBLING IN LAST 12 MONTHS – C20=1]
C21 – In the last 12 months, have you spent money on ONLINE gambling?

1. Yes
2. No

999. [Don't know / No answer]

[ASK IF GAMBLING IN LAST 12 MONTHS – C20=1]
C22 – Thinking about gambling, how often would you say you spend money on it?

1. Two or more times per week
2. Once a week
3. Less than once a week but more than once a month
4. Once a month
5. Every 2–3 months
6. Once or twice a year

999. [Don't know / No answer]

[ASK IF GAMBLING IN LAST 12 MONTHS – C20=1]
C23 – Could you please indicate how much money you spent on gambling in the last month?

1. I did not gamble in the last month
2. Up to €5
3. €5 or more, but less than €10
4. €10 or more, but less than €20
5. €20 or more, but less than €50
6. €50 or more, but less than €100
7. €100 or more

999.

SECTION IV – INDIVIDUAL SOCIAL NETWORK

Introduction

We will now ask you to reconstruct the contacts with the people you usually meet. This is of interest to the research because one of its aims is to investigate the diffusion of health-related behaviours within the population.
The names you will provide will be anonymised, and the data will be processed in such a way that it will not be possible for the researchers to trace back their identity once the survey has been completed.

4.1 INTRA-MUNICIPAL NETWORK

A1 – Excluding the people who live with you, could you please indicate the persons who reside in your municipality of residence, whose first and last names you know, and with whom you usually meet and converse on a regular basis? Please do not include people you meet only occasionally or rarely.

ONLY IF CLARIFICATION IS REQUESTED: By "usually meet and converse on a regular basis" we mean, for example, having a conversation lasting at least 30 minutes once a week, or a conversation of 10–20 minutes several times a week.

NOTES FOR INTERVIEWER:

- The list of residents with their individual codes will be used. In case of homonyms, verify by date of birth or address.
- The list must never be shown to the respondent.
- If clarification is requested, explain that only an anonymised numerical code will be recorded.
- If the person mentioned does not appear in the residents' list, they will be treated as an *extra-municipal case* (see Section B).

Table of Contacts

Unique Code	A1.1	A1.2	A1.3	A1.4	A1.5
1					
2					
3					
4					
5					
6					
7					
…					

For each person indicated:
A1.1 – What kind of relationship do you have with this person?

1. Relative
2. Friend
3. Colleague / work relationship
4. Acquaintance
5. [Other: specify _ _ _]

999. [Don't know / No answer]

A1.2 – For how long have you known this person?

1. Less than 1 year
2. 1–5 years
3. 6–10 years
4. More than 10 years
9. [Prefer not to answer]

A1.3 – How strong would you say your relationship with this person is?

1. Very strong
2. Fairly strong
3. Neither strong nor weak
4. Fairly weak
5. Very weak

999. [Don't know / No answer]

A1.4 – In which context do you usually meet this person? (multiple answers possible)

1. At home
2. Workplace
3. School / university
4. Sporting activity (e.g. running, football, volleyball, cycling, etc.)
5. For lunch or dinner
6. Bar / café / pub
7. Restaurant / trattoria
8. Leisure or recreational activity (e.g. walking, cycling, playing cards, bowling club, disco, etc.)
9. While shopping / at the market
10. [Other: specify _ _ _]

999. [Don't know / No answer]

A1.5 – Do you ever discuss with this person topics related to health, or behaviours and habits that may have consequences for health? For example: diets, physical activity, doctors, medicines, illnesses, health advice.

1. Yes, often
2. Yes, sometimes
3. Never

999.

4.1 INTRA-MUNICIPAL NETWORK

A2 – Excluding the people who live with you, could you please indicate the persons in your municipality, whose first and last names you know, with whom you have the habit of regularly talking or communicating by telephone or digital means (such as WhatsApp or Facebook)? Please exclude occasional or very brief calls or communications, as well as group conversations. You may also indicate the same persons already mentioned among those you usually meet in person.

ONLY IF CLARIFICATION IS REQUESTED: By "habit of regularly talking or communicating by telephone or digital means" we mean, for example, having a conversation lasting at least 5 minutes at least several times a week, or frequent communication via voice or text messages several times a week.

NOTES FOR INTERVIEWER:

- The list of residents with their individual codes will be used. In case of homonyms, verify by date of birth or address.
- The list must never be shown to the respondent.
- If clarification is requested, explain that only an anonymised numerical code will be recorded.
- If the person mentioned does not appear in the residents' list, they will be treated as an *extra-municipal case* (see Section B).
-

Table of Contacts

Unique Code	A2.1	A2.2	A2.3	A2.4
1				
2				
3				
4				
5				
6				
…				

[ONLY IF DIFFERENT FROM A1]
For each person indicated:

A2.1 – What kind of relationship do you have with this person?

1. Relative
2. Friend
3. Colleague / work relationship
4. Acquaintance
5. [Other: specify _ _ _]

999. [Don't know / No answer]

A2.2 – For how long have you known this person?

1. Less than 1 year
2. 1–5 years
3. 6–10 years
4. More than 10 years
9. [Prefer not to answer]

A2.3 – How strong would you say your relationship with this person is?

1. Very strong
2. Fairly strong
3. Neither strong nor weak
4. Fairly weak
5. Very weak
999. [Don't know / No answer]

A2.4 – Do you ever discuss with this person topics related to health, or behaviours and habits that may have consequences for health? For example: diets, physical activity, doctors, medicines, illnesses, health advice.

1. Yes, often
2. Yes, sometimes
3. Never
4. I don't remember
999. [Don't know / No answer]

A3 – Excluding the people who live with you and those already mentioned, are there other persons in your municipality, whose first and last names you know, with whom you keep in contact (even occasionally), and with whom you talk (by any means, including social networks) about topics related to health? For example: diets, physical activity, doctors, medicines, illnesses, health advice.

INTERVIEWER NOTE:

- At this question, it is possible to record also the following health professionals if spontaneously mentioned by the respondent: **doctor, pharmacist, dentist**.
- The list of residents with their individual codes will be used. In case of homonyms, verify by date of birth or address.
- The list must never be shown to the respondent.
- If clarification is requested, explain that only an anonymised numerical code will be recorded.
- Add to the list the health professionals present in XXX : **doctor, pharmacist, dentist**.
- If the person is not in the residents' list, even if de facto resident in XXX , they must not be interviewed and will not appear in Section B.
- Only unique codes should be recorded, without further questions.
- Add codes of some key health professionals (if not residents of XXX: doctor, pharmacist, dentist – see manual, Question A3).

Table – Unique Codes

	**Unique Code**
1	
2	
3	
4	
5	
…	

A4 – Which public places do you usually attend in your municipality? *(Multiple answers possible – wait for spontaneous response from respondent)*

1. Town Hall
2. Church Square
3. Park in Via XXX
4. Parish
5. Bar 1
6. Bar 2
7. Bar 3
8. Bar 4
9. Oratory
10. Sports centre
11. School
12. Bowling club
13. Market
14. Hairdresser
15. Other (specify _ _ _ _ _)

999. [Don’t know / No answer]

4.2 EXTRA-MUNICIPAL NETWORK

We will now ask you to name your extra-municipal contacts [i.e. those not included in the Residents' List], using a first name, or perhaps the initial of the surname, or a nickname, so that we can distinguish one person from another. All names will be kept strictly confidential, as explained in the consent form. Please make sure to provide names or nicknames of individuals, not categories of people (for example, "Andrea" is acceptable, but "the company where I work" is not).

B1 – Could you please indicate the characteristics of the persons who do not reside in your municipality [or who are not included in the Residents' List], with whom you most often have the habit of meeting and conversing on a regular basis (beyond casual or sporadic encounters)?
Up to a maximum of 8 persons.
ONLY IF CLARIFICATION IS REQUESTED: By "habit of meeting and conversing on a regular basis" we mean, for example, having a conversation lasting at least 30 minutes once a week, or a usual conversation of 10–20 minutes several times a week.

For each person indicated:

	**1**	**2**	**3**	**4**	**5**	**6**	**7**	**8**
**B1.01** Name (or nickname)	…	…	…	…	…	…	…	…
**B1.02** Sex or Gender	1. Male / 2. Female / [3. Other]							
**B1.03** Approximate age	1. <18 / 2. 18–29 / 3. 30–39 / 4. 40–49 / 5. 50–59 / 6. 60–69 / 7. 70+ / [999. Don't know / No answer]							
**B1.04** Highest level of education	1. University degree / 2. Secondary diploma / 3. Lower secondary / 4. Primary / 5. No qualification / [999. Don't know / No answer]							
**B1.05** Employment status	1. Self-employed / 2. Employee / 3. Unemployed / 4. Unpaid domestic work / 5. Retired / 6. Student / 7. Unable to work / 8. Other / [999. Don't know / No answer]							
**B1.06** Place of residence	1. In XXX / 2. In a nearby municipality (less than 30 minutes away, by any means) / 3. In a distant municipality (more than 30 minutes away, by any means) / [999. Don't know / No answer]							
**B1.07** Relationship	1. Relative / 2. Friend / 3. Colleague / work relationship / 4. Acquaintance / 5. [Other: specify] / [999. Don't know / No answer]							
**B1.08** How long have	1. Less than 1 year / 2. 1–5 years / 3. 6–10 years							

	1	2	3	4	5	6	7	8
you known this person?	/ 4. More than 10 years / 9. [Prefer not to answer]							
**B1.09** Strength of relationship	1. Very strong / 2. Fairly strong / 3. Neither strong nor weak / 4. Fairly weak / 5. Very weak / [999. Don’t know / No answer]							
**B1.10** [B1 only] In which context do you usually meet this person? (multiple answers possible)	01. At home / 02. Workplace / 03. School or university / 04. Sporting activity (running, football, volleyball, cycling, etc.) / 05. For lunch or dinner / 06. Bar / café / pub / 07. Restaurant / trattoria / 08. Leisure or recreational activity (walking, cycling, playing cards, bowling club, disco, etc.) / 09. While shopping / at the market / 10. [Other: specify] / 999. [Don’t know / No answer]							
**B1.11** Do you ever discuss with this person topics related to health, or behaviours and habits that may have consequences for health?	1. Yes, often / 2. Yes, sometimes / 3. Never / [999. Don’t know / No answer]							
**B1.12** To your knowledge, is this person overweight?	1. Yes / 2. No / [999. Don’t know / No answer]							
**B1.13** To your knowledge, is this person a smoker?	1. Yes / 2. No / [999. Don’t know / No answer]							
**B1.14** To your knowledge, does this person frequently consume alcoholic beverages?	1. Yes / 2. No / [999. Don’t know / No answer]							

B2 – Could you please indicate the characteristics of the persons who do not reside in your municipality [or who are not included in the Residents' List], with whom you most often have the habit of regularly talking or communicating by telephone or digital means (such as WhatsApp or Facebook)?

Please exclude occasional or very brief calls or communications, as well as group conversations. If you have already mentioned them previously, please indicate only the name or nickname of the person already listed. Up to a maximum of 8 persons.

ONLY IF CLARIFICATION IS REQUESTED: By "habit of regularly talking or communicating by telephone or digital means" we mean, for example, having a conversation lasting at least 5 minutes at least several times a week, or frequent communication via voice or text messages several times a week.

[ASK ONLY IF DIFFERENT FROM CONTACTS INDICATED IN B1] *(See previous table, excluding question B2.10 on meeting places)*

4.3 DIGITAL MEDIA NETWORK

N1 – Do you use the following platforms to communicate/interact with relatives, friends, colleagues or acquaintances (excluding strangers)? *(Multiple answers possible)*

- Facebook – Yes / No
- WhatsApp – Yes / No
- TikTok – Yes / No
- Instagram – Yes / No
- X (formerly Twitter) – Yes / No
- Telegram – Yes / No
- [Other: specify _ _ _]

[ASK ONLY IF FACEBOOK USER – N1=1]

N2 – If you use Facebook, could you please indicate approximately how many friends you have on Facebook?

1. Fewer than 20
2. 21–50
3. 51–100
4. 101–130
5. 131–170
6. 171–300
7. More than 300

999. [Don't know / No answer]

Request for Authorisation for Possible Follow-up Interview

We thank you very much for your collaboration. We would like to ask you one more favour. The research foresees that a group of respondents may be contacted again for an in-depth interview on health-related behaviours. We therefore ask for your availability to be contacted again for a conversation of about one hour.
The interview will be used in an entirely anonymous form, making it impossible for others to identify you. For this second interview, a token of €30 in shopping vouchers will be offered.

Do you authorise being re-contacted for an in-depth interview?

- Authorise
- Do not authorise

Contact Details

Telephone number

- Provides: ___________________
- Does not provide a number

Email address

- Provides: ___________________
- Does not provide an email address

Interviewer Notes

Please also provide an open field **"Observations/Notes on the Interview"** where the interviewer may record any comments or notes regarding the interview.
